# Editorial: The Role of Nuclear Molecules in the Pathogenesis of Autoimmune Disease

**DOI:** 10.3389/fimmu.2021.737923

**Published:** 2021-08-05

**Authors:** David S. Pisetsky, Reinhard Voll, Edit I. Buzas

**Affiliations:** ^1^Department of Medicine and Immunology, Duke University Medical Center, Durham, NC, United States; ^2^Medical Research Service, Durham Veterans Administration Medical Center, Durham, NC, United States; ^3^Department of Rheumatology and Clinical Immunology, Medical Center – University of Freiburg, Faculty of Medicine, University of Freiburg, Freiburg, Germany; ^4^HCEMM-SU and ELKH-SE Immune-Proteogenomics Research Extracellular Vesicle Research Group, Semmelweis University, Budapest, Hungary

**Keywords:** DNA, HMGB1, NETosis, autoimmunity, systemic lupus, extracellular vesicles

Nuclear molecules are a diverse set of macromolecules whose designation as nuclear derives from their location rather than chemistry or function. These molecules include DNA, RNA and proteins although, in general, nuclear molecules exist as complexes of proteins and nucleic acids inside the cell. The focus on the nucleus as a defining feature of these molecules, while clearly correct, may nevertheless underestimate the importance of the translocation events that nuclear molecules undergo to serve their function. Thus, while DNA, RNA and proteins are major chemical constituents of the nucleus, their location is neither uniform nor fixed. As a group, these molecules can all migrate around the cell and variably appear in the nucleus and cytoplasm. Furthermore, they can exit the cell to enter the blood where they can display powerful immunological activities, seemingly unrelated to their nuclear function, and can drive the pathogenesis of autoimmunity.

The articles in this Research Topic, “The Role of Nuclear Molecules in the Pathogenesis of Autoimmune Disease,” provide an exciting perspective on molecules for which the nucleus is just one site of action. In this editorial, we will provide an overview of the field and indicate the areas in which the articles provide new understanding. While the field is rapidly evolving, nevertheless, the articles stand as important contributions that will help guide future directions. The excitement about this series in many respects reflects the early stage of the research. This is all new territory and key questions about the origin of this movement remain largely unanswered: is translocation to the extracellular space a normal physiological process for information transfer or signaling? Or, is this movement evidence for pathophysiology, the end-product of cellular damage and death, with nuclear molecules going rogue once outside the confines of the cell? Have extracellular activities shaped the evolution of these chemical structures or has the host adapted to the structures encountered?

Among nuclear molecules with prominent extracellular expression in autoimmune and inflammatory disease, DNA was identified in the blood many years ago with the study of systemic lupus erythematosus (SLE) providing compelling evidence for the critical role of extracellular DNA in disease pathogenesis (Duvvuri and Lood; Mustelin et al.; Soni and Reizis). SLE is a prototypic autoimmune disease characterized by antibodies to nuclear molecules (antinuclear antibodies or ANAs). These target antigens include DNA, RNA and protein-nucleic acid complexes. Of ANAs, anti-DNA antibodies are unique in that they are markers for diagnosis, classification and disease activity.

In the pathogenesis of SLE, immune complexes containing nuclear molecules play a key role in the inflammation characteristic of this disease, especially in the kidney (Rekvig). As early studies showed, levels of anti-DNA antibodies can rise with disease activity, often in association with depression of complement, indicative of immune complex formation. While the formation of immune complexes consisting of DNA and anti-DNA does not necessitate a change in levels of extracellular DNA, in fact, DNA levels also rise concomitant with the increases in anti-DNA. The basis of this increase has long been a source of fascination and, indeed, is one of the impetuses to study extracellular DNA, truly an epicenter of SLE (Soni and Reizis).

The term extracellular specifies a locale, but it does not specify a chemical structure. In view of its interaction with histones and non-nuclear histone proteins in the cell, extracellular DNA is likely to be bound with histones in the form of nucleosomes or chromatin. The physical-chemical properties of nucleosomal DNA differ from that of free DNA including susceptibility to different extracellular nucleases as shown in studies on the biology of DNase 1L3 (1 like 3) (Soni and Reizis). Extracellular chromatin may be a more apt terminology for extracellular DNA but, in the development of novel biomarkers, the focus is DNA and not the histones since DNA can be interrogated in much greater depth to identify its source (e.g., malignant cells).

In addition to its association with proteins, DNA in the extracellular space can exist as a free or soluble form as well as a particulate. Extracellular vesicles, which include microvesicles (also termed microparticles) and other vesicle types, can be generated during the steady state, cell activation or cell death (Burbano et al.; Rasmussen et al.). Extracellular vesicles may contain DNA, histones and HMGB1 (high mobility group box 1) among other nuclear and cytoplasmic molecules and have potent activities that can promote inflammation as well as thrombosis. Apoptosis is an important source of extracellular nuclear molecules in a particle form. It is unknown, however, whether the formation of vesicles is a simple physical-chemical consequence of certain forms of cell death or if vesicle formation has evolved to promote phagocytic uptake or clearance. The transmission of danger signals is another potential action of particles that may have evolved.

NETosis is an additional process that can generate extracellular DNA that includes mitochondrial DNA which differs structurally from nuclear DNA in its lack of methylation of CpG motifs. Mitochondrial DNA also has the propensity for oxidation which can further increase its immunostimulatory potential. NETs (neutrophil extracellular traps) are an elaborate form of extracellular DNA which can be extruded from neutrophils and other immune cells as a defense strategy. Given its dense, mesh-like structure, a NET can trap bacteria, viruses or fungi, with killing accomplished by anti-bacterial proteins such as histones and enzymes contributed by neutrophils. Among extracellular forms of DNA, NETs can form large aggregates that bind other molecules and modulate their activities (Knopf et al.), with degradation of these structures also important in determining pathological states such as thrombosis, immune complex formation and autoimmunity. The assay of NETs is important in the study of immunopathogenesis although quantitation remains a challenge (van Brenda et al.).

Along with the recognition of the movement of nuclear molecules to the extracellular space came studies that DNA, RNA, histones and HMGB1 all have intrinsic immunological activity and could stimulate both toll-like receptors (TLRs) and non-TLR sensors. In particular, HMGB1 has diverse activities that mediate events in many autoimmune and inflammatory diseases (Gorgulho et al.; Sowinska et al.). These molecules may act alone or in concert as complexes such as chromatin which contain DNA and proteins (Ribon et al.). In this regard, bacterial DNA as well as host mammalian DNA can appear in the blood reflecting the presence of bacterial organisms and their growth as biofilms (Qiu et al.).

While DNA and RNA have immune activity, the location for the contact of nucleic acid sensors is critical. For stimulation of immunity, the contacts with nucleic acid sensors occur in the cytoplasm, including the endosomal compartment. In the context of diseases like SLE, the uptake of nucleic acids into cells of the innate immune system provides a source of either DNA or RNA to interact with the sensors and stimulate production of cytokines such as type 1 interferon. In this activation, the immune complexes provide a conduit of nucleic acids into different cell compartments.

In SLE, internal nucleic acid sensors can be activated by either extracellular DNA or RNA that enters the cell as immune complexes. An important function of these sensors is to promote host defense against intracellular infection whether virus, bacteria or fungi. Indeed, while models of host defense often focus on the extracellular space and generation of protective antibodies, inhibition of pathogen proliferation occurs intracellularly, with foreign nucleic acids as key signals to activate host defense. These sensors can also be triggered by nucleic acids that are aberrantly present in the cytoplasm because of cell stress as well as abnormalities in the DNase and RNase enzymes that keep the cytoplasm free from nucleic acids.

For DNA, the cytoplasm along with the nucleus is an important location for DNA, with mitochondria having their own DNA to encode certain mitochondrial molecules. Unlike nuclear DNA, mitochondrial DNA does not exist in a nucleosomal structure and histones are not present. Although mitochondrial DNA can be safely ensconced in a membrane-bound structure, it can be released into the cytoplasm with mitochondrial damage; mitochondrial DNA can also appear in the blood along with nuclear DNA during cell death. Importantly, mitochondrial DNA has immune activity because of its resemblance to bacterial DNA. It is of interest that patients with SLE produce antibodies to mitochondrial RNA while antibodies to other sources of RNA appear uncommon (Becker et al.). Perhaps, mitochondrial RNA may differ in immunogenicity because of its structure or association with other immunostimulatory molecules present in the mitochondria.

The immune properties of extracellular DNA and RNA and their sensing systems have been one of the most exciting and productive areas of immunology research in the past few years as the papers in this series indicate. These studies have also revealed the importance of new forms of RNA such as lncRNA (long non-coding RNA) (Zhang et al.). Along with intriguing new data have come conceptual advances to understand host defense. To name a few, we would note the following ideas that have changed the paradigms in immunology: immune complexes provide a mechanism to transmit DNA and RNA to internal nucleic acid sensors; cell death is a key element of host defense; and defects in DNase and RNase enzymes can lead to autoimmunity. Another conceptual as well as technical advance relates to the use of proteomics to find other cell constituents that can serve as biomarkers in the blood (Idborg et al.).

While this Research Topic focuses on the extracellular location of nuclear molecules, the mechanisms described all relate to the mobility of nuclear molecules and the translocation events they can undergo. These events start with a translocation from the inside of a cell to its outside. A subsequent translocation event moves RNA and DNA from the outside of one cell back inside of another one. Once inside another cell, the DNA and RNA can access sensing systems that drive inflammation and cell death. From this perspective comes a host of novel ideas for developing new therapies: scavenging, degrading of binding extracellular nuclear molecules; inhibiting their sensors; and blocking the downstream mediators induced.

The story on extracellular nuclear molecules is just beginning and, as these reviews and original papers illustrate so insightfully, the nucleus is just one location where these molecules can act. Future studies will hopefully discover new ways to block their action once they have left the cell and provide new approaches to treat the broad range of autoimmune and inflammatory diseases in more effective and safer ways.

## Author Contributions

All authors contributed to the article and approved the submitted version.

## Funding

DSP is supported by a VA Merit Review grant as well as an NIH grant (1RO1AR073935).

## Conflict of Interest

The authors declare that the research was conducted in the absence of any commercial or financial relationships that could be construed as a potential conflict of interest.

## Publisher’s Note

All claims expressed in this article are solely those of the authors and do not necessarily represent those of their affiliated organizations, or those of the publisher, the editors and the reviewers. Any product that may be evaluated in this article, or claim that may be made by its manufacturer, is not guaranteed or endorsed by the publisher.

